# Do porcine Sertoli cells represent an opportunity for Duchenne muscular dystrophy?

**DOI:** 10.1111/cpr.12599

**Published:** 2019-03-26

**Authors:** Sara Chiappalupi, Laura Salvadori, Giovanni Luca, Francesca Riuzzi, Riccardo Calafiore, Rosario Donato, Guglielmo Sorci

**Affiliations:** ^1^ Department of Experimental Medicine University of Perugia Perugia Italy; ^2^ Interuniversity Institute of Myology (IIM) Perugia Italy; ^3^ Department of Medicine University of Perugia Perugia Italy; ^4^ Centro Universitario di Ricerca sulla Genomica Funzionale University of Perugia Perugia Italy

**Keywords:** Duchenne muscular dystrophy, encapsulation, muscle inflammation, Sertoli cell, therapeutic approaches

## Abstract

Sertoli cells (SeC) are responsible for the immunoprivileged status of the testis thanks to which allogeneic or xenogeneic engraftments can survive without pharmacological immune suppression if co‐injected with SeC. This peculiar ability of SeC is dependent on secretion of a plethora of factors including maturation factors, hormones, growth factors, cytokines and immunomodulatory factors. The anti‐inflammatory and trophic properties of SeC have been largely exploited in several experimental models of diseases, diabetes being the most studied. Duchenne muscular dystrophy (DMD) is a lethal X‐linked recessive pathology in which lack of functional dystrophin leads to progressive muscle degeneration culminating in loss of locomotion and premature death. Despite a huge effort to find a cure, DMD patients are currently treated with anti‐inflammatory steroids. Recently, encapsulated porcine SeC (MC‐SeC) have been injected ip in the absence of immunosuppression in an animal model of DMD resulting in reduction of muscle inflammation and amelioration of muscle morphology and functionality, thus opening an additional avenue in the treatment of DMD. The novel protocol is endowed with the advantage of being potentially applicable to all the cohort of DMD patients regardless of the mutation. This mini‐review addresses several issues linked to the possible use of MC‐SeC injected ip in dystrophic people.

## 1 INTRODUCTION

Sertoli cells (SeC) are major component of the seminiferous tubules of the testis where they contribute to the development of germ cells and protect germ cells from the attack by the host immune system.^1^, ^2^ Indeed, newly synthesized markers on germ cell surface would be recognized as non‐self by the immune system, as this latter becomes mature before spermatogenesis starts. SeC exert their double role (a) by creating a physical barrier (the blood‐testis barrier, BTB) made of adjacent SeC linked together with tight junctions, isolating the lumen of seminiferous tubules from the interstitial fluid, and (b) by secreting a plethora of trophic and immunomodulatory factors.^3^, ^4^ This latter ability of SeC has prompted researchers to use them in many experimental models of diseases in which supplying trophic factors, abating inflammation or modulating the immune system activity might result in reversion or attenuation of the pathology.^2^, ^5^

## 2 SERTOLI CELLS

The immunoprivileged status of the testis is long known. In 1767, during his studies on transplantation, John Hunter observed that rooster testes transplanted into the abdominal cavity of a hen maintained normal structure over time.^6^ Subsequent investigation identified SeC as the cell type mainly responsible for the immunological properties of the testis. Indeed, allogeneic and/or xenogeneic pancreatic islets,^7^, ^8^ adrenal chromaffin cells^9^ and dopaminergic neurons^10^ were successfully protected by SeC in the absence of pharmacological immune suppression in different experimental models. Similarly, skin^11^ and heart^12^ grafts showed prolonged survival when co‐injected with SeC. Moreover, grafts of SeC alone exerted trophic effects in the central nervous system in animal models of Huntington’s disease and amyotrophic lateral sclerosis.^13^, ^14^

Intriguing results about SeC derive from their use as encapsulated cells in several pre‐clinical studies. Alginate‐based microcapsules containing SeC (MC‐SeC) have been successfully employed as a single intraperitoneal (ip) injection in experimental models of type 1 and type 2 diabetes, acute hepatic failure, skin graft and Huntington’s disease.^15^, ^16^, ^17^, ^18^, ^19^, ^20^, ^21^ In an animal model reproducing the human Laron syndrome, in which mutations in the growth hormone receptor (GHR) lead to reduced production of IGF‐1 and subsequent dwarfism, intraperitoneally injected MC‐SeC promoted body growth through the release of IGF‐1 into the circulation.^22^

The trophic and immunomodulatory properties of SeC are dependent on the complex SeC secretory activity resulting in a cocktail of factors whose formulation is difficult to dissect, being affected by the biological status of the cells and likely by environmental cues. While the composition of SeC’ secretory product still awaits to be identified, it includes maturation factors, hormones, growth factors, cytokines and immunomodulatory factors (Table 1).

**Table 1:** Factors known to be secreted by SeC

Maturation factors and hormones	Activins^116^ Dhh^117^	Oestrogens^118^ Inhibins^116^	KL/SCF^119^, ^120^ MIS/AMH^121^
Growth factors and cytokines	BDNF^122^ bFGF^123^ BP4^124^ EGF^125^ GDNF^126^ Heregulin‐β1^94^	IFN‐γ^127^ IGF‐1^128^, ^129^ IGF‐2^130^ IL‐1, IL‐6^24^, ^131^, ^132^ SCSGF^133^ NT‐3^122^, ^134^	PDGF^135^ SGP‐1/Prosaposin, SGP‐2^136^, ^137^ TGF‐α, TGF‐β^138^, ^139^ VEGF^140^
Immunomodulatory factors	Activin A^116^ BCL‐w^141^ Clusterin^142^ Complement cascade inhibitors^26^	FasL^143^, ^144^ IDO^15^ IL‐2 suppressor factors^23^ JAG1^29^	MIF^145^ Serpins^27^ TGF‐β^139^ Transferrin^146^, ^147^

AMH, anti‐Müllerian hormone; BDNF, brain‐derived neurotrophic factor; bFGF, basic fibroblast growth factor; BMP4, bone morphogenetic protein 4; Dhh, desert hedgehog; EGF, epidermal growth factor; FasL, Fas ligand; GDNF, glial cell–derived neurotrophic factor; IDO, indoleamine 2,3‐dioxygenase; IFN, interferon; IGF, insulin‐like growth factor; IL, interleukin; JAG1, soluble JAGGED1; KL, kit ligand; MIF, macrophage inhibitory factor; MIS, Müllerian‐inhibiting substance; NT, neurotrophin; PDGF, platelet‐derived growth factor; SCF, stem cell factor; SCSGF, SeC‐secreted growth factor; SGP, sulphated glycoprotein; TGF, transforming growth factor; VEGF, vascular endothelial growth factor.

However, the immunomodulatory effect of SeC is obtained by a multimodal mechanism. SeC secrete (still unidentified) factors thatblock T lymphocyte proliferation and interleukin (IL)‐2 production,^23^, ^24^ and SeC induce apoptosis of lymphocytes mediated by the interaction of FasL expressed on their surface and Fas receptor (CD95) expressed on T cells.^25^ SeC secrete inhibitors of the complement cascade and granzyme, a cytolytic molecule released by cytotoxic T cells.^26^, ^27^ Moreover, the secretion of specific factors, such as TGF‐β, IDO (indoleamine 2,3‐dioxygenase), activin A and JAG1, concurs to immunomodulation favouring the emergence of tolerogenic cells, including M2 (anti‐inflammatory) macrophages, and Th2 and Tregs.^15^, ^28^, ^29^

## 3 DUCHENNE MUSCULAR DYSTROPHY AND RELATED THERAPEUTIC APPROACHES

Duchenne muscular dystrophy (DMD) is an X‐linked recessive disease due to mutations in the dystrophin gene (*DMD*), the biggest gene of the human genome for which about 4700 different mutations have been reported.^30^, ^31^, ^32^, ^33^ Dystrophin is an essential component of the dystrophin‐associated protein complex (DAPC), a multiprotein complex located at the sarcolemma and responsible for the mechanical link between the intracellular cytoskeleton and the extracellular matrix; dystrophin ensures the structural and functional integrity of myofibres during contraction. *DMD* gene mutations translating into absence of dystrophin or expression of functionally inefficient protein lead to the Duchenne phenotype, in which loss of the integrity of DAPC causes myofibre degeneration and progressive loss of muscle efficiency, wheelchair dependency before teenage years, and premature death by cardiac and respiratory failure.^31^, ^34^ Morphologically, DMD muscles are characterized by infiltration with immune cells and chronic activation of inflammatory signalling pathways due to continuous degeneration/regeneration cycles, with the final result that fibrous and fatty tissues progressively overtake functional myofibres.^35^

Therapeutic approaches to DMD have been experiencing multiple obstacles against their success (Table 2). Firstly, *DMD* gene is a too large gene (2.4 Mb and 79 exons, corresponding to about 0.1% of the human genome)^30^ to be delivered using classical recombinant adeno‐associated viruses (rAAVs), which has led to investigation of the use of parts of the gene translating into shorter but still efficient proteins, that is, mini‐ and micro‐dystrophins.^36^, ^37^, ^38^, ^39^, ^40^ Although AAV vectors carrying mini‐dystrophin have given encouraging results in *mdx *mice (the most used experimental model for DMD) and GRMD (golden retriever muscular dystrophy) dogs,^41^, ^42^, ^43^ phase I clinical trial revealed only a limited dystrophin expression and irrelevant muscle improvements due to the host immune response.^44^

**Table 2:** Advantages and disadvantages of the main therapeutic approaches to DMD

Approach	Advantages	Disadvantages	References
Gene therapy	Potential rescue of functional dystrophin in cardiac and skeletal muscles Independent from *DMD* gene mutation AAV vectors are already approved for other pathologies	Need to use truncated forms of dystrophin due to the high size of *DMD* gene Requirement of high doses of vectors Possible immune reaction against vectors Potential need for immunosuppressive therapy	[37, 38, 39, 40]
Exon skipping	Restoration of expression of partially functional dystrophin Some AON are well tolerated Eteplirsen received conditional approval by USA FDA	*DMD* gene mutation‐dependent Scarce tissue uptake Large doses and repeated injections are required Significant side effects are reported in some cases Controversial efficacy	[47, 48, 49]
Cell therapy	Restoration of functional dystrophin Possibility to reprogramme adult somatic cells to iPSC Possibility to correct mutations ex vivo in patient cells Low risk of immune reaction in autologous transplantations	Short lifespan and low migration ability of injected cells Immune reaction when cells come from healthy donors Requirement of immunosuppression in allogeneic transplantations	[47, 55, 56, 57]
Utrophin induction	Independent from *DMD* gene mutation Oral administration Well‐tolerated compounds No requirement of immunosuppressive treatment	Ezutromid (SMT C1100) failed to reach its objectives	[53, 69]

AAV, adeno‐associated viruses; AON, antisense oligonucleotides; FDA, Food and Drug Administration; iPSC, induced pluripotent stem cells.

Exon skipping is an approach to overcome specific regions with deletions, duplications or small mutations in the *DMD *gene pointing to recovery of the reading frame and production of truncated but functional forms of dystrophin, and translating into a switch from the DMD pathology to the milder phenotype known as Becker muscular dystrophy (BMD).^45^, ^46^ Specifically designed antisense oligonucleotides (AON), which are 20‐30 nucleotides in length, are used to obtain skipping of different exons resulting in truncated but in‐frame transcripts.^47^, ^48^, ^49^ The modified AON, 2′‐*O*‐methyl‐p phosphorothioate oligonucleotides (PS) and phosphorodiamidate morpholino oligomers (PMO) have shown high stability and efficacy, and low toxicity. One major limit of AON is that they are only useful for DMD patients with specific mutations and not applicable to the remaining cohort of patients. The PS, Drisapersen and the more promising PMO, Eteplirsen, both of which are in clinical trials, are specific for skipping of exon 51, which applies to 14% of patients, that is, the largest cohort of DMD subjects who may benefit from single exon skipping.^39^, ^50^ Other limitations inherent to the use of AON are represented by their scarce tissue uptake and low rescue of dystrophin expression in muscles.

Cell therapy represents a second front of therapeutic approaches to DMD. It tries to use different cell types (especially, satellite cells/myoblasts, mesoangioblasts and induced pluripotent stem cells [iPSC]) from healthy donors or genetically engineered cells from the patients themselves to obtain the re‐expression of dystrophin in muscle tissue and recovery of muscle performance.^51^, ^52^, ^53^, ^54^ Cells obtained from patients are corrected ex vivo and re‐implanted in the donors, whereas cells from healthy donors are used in allogeneic transplantations in dystrophic patients.^55^ This kind of approach is finding limits in the low survival of injected cells and their inability to migrate for long distances, so that repeated local injections are required.^56^ The reasons why injected cells show low survival and scarce ability to migrate are not fully understood, but deficiency of specific growth factors might play an important role. Immune rejection of transplanted cells is another relevant concern in the case of allogeneic approaches, which require concomitant pharmacological immunosuppression.^57^

Healthy human satellite cells and myoblasts (ie, muscle precursor cells) induce dystrophin expression in DMD patients to a certain extent when injected intramuscularly^58^; a phase I/II clinical trial (NCT02196467) is still ongoing. iPSC are somatic cells (including fibroblasts, hepatocytes, pancreatic beta cells, lymphocytes and neural progenitor cells) reprogrammed in vitro to a pluripotent state by ectopic expression of Oct4 and Sox2 in combination with either Klf4 and c‐Myc or Lin28 and Nanog.^59^ iPSC show similarity to embryonic stem cells, can be directed to mesenchymal differentiation, and patient‐derived iPSC can be genetically engineered for potential autologous therapies. However, iPSC have found employment only in up to pre‐clinical studies so far.^60^ Mesoangioblasts (ie, cells associated with the walls of large vessels) represent one of the most promising cell types for cell therapy in DMD patients. Mesoangioblasts are endowed with myogenic potential and ability to cross the blood vessel wall, and their use has resulted in improvement of muscle morphology in several experimental models of muscular dystrophy.^45^, ^61^, ^62^ Intra‐arterial injection of allogeneic human mesoangioblasts isolated from adult skeletal muscle is currently under phase I clinical trial (EudraCT #2011‐000176‐33).

The existence of the dystrophin paralogue, utrophin, has fostered another approach to rescue homeostasis in the muscles of DMD patients. Utrophin shares a very high degree of sequence identity with dystrophin and even associates with members of the DAPC, thus mimicking the role of dystrophin in dystrophin‐negative myofibres.^30^, ^63^ In healthy adult muscle fibres, dystrophin and utrophin show different expression patterns, with dystrophin being expressed along the entire sarcolemma and utrophin confined to the myotendinous and the neuromuscular junctions (NMJs).^64^, ^65^ However, utrophin is expressed at high levels at the sarcolemma during development, when dystrophin is not expressed yet.^66^ Indeed, necrosis of *mdx* limb muscles begins only when the high neonatal levels of utrophin become reduced to adult levels.^64^ Since forced expression of utrophin in dystrophic myofibres can restore assembly of DAPC members at the sarcolemma and prevent the dystrophic pathology,^67^, ^68^ up‐regulation of utrophin in muscles represents a still active field of investigation in DMD treatment.^69^ In this regard, the small molecule, SMT C1100 (Ezutromid), which has shown promising results in a phase I clinical trial,^70^ was stopped after a phase II clinical trial (NCT02858362) since it failed to reach its primary and secondary objectives.

Several alternative approaches to treat DMD are currently under investigation (Table 3). They include use of the histone deacetylase (HDAC) inhibitor, Givinostat^71^, ^72^, ^73^; the phosphodiesterase‐5 (PDE5) inhibitors, Tadalafil and Sildenafil^74^, ^75^, ^76^; the benzoquinone, Idebenone (Catena/Raxone)^77^, ^78^, ^79^; the aminoglycoside, Ataluren (Translarna, former PTC124).^80^, ^81^ The anti‐fibrotic molecule, Halofuginone (HT‐100),^82^ and the anti‐myostatin monoclonal antibody, Domagrozumab,^83^, ^84^ have been blocked in phase II clinical trial due to death of a patient receiving the highest dose and no significant therapeutic effects, respectively. While treatment with Ataluren points to re‐expression of dystrophin in muscles, the majority of alternative approaches are aimed at restraining pathogenic mechanisms secondary to lack of dystrophin, particularly muscle inflammation and fibrosis.

**Table 3:** Principal currently ongoing alternative approaches to treat DMD

Drug	Description/Activity	Effects	Limitations	Clinical trial	References
Ataluren (PTC124) *PTC* *Therapeutics*	Small chemical compound that induces ribosomal read‐through of premature stop codons	Restoration of expression of full‐length dystrophin	Use limited to patients with nonsense mutations (nmDMD)	Phase III completed Conditional approval in Europe	[63, 64]
Givinostat *Italfarmaco*	Inhibitor of HDAC (enzymes that prevent gene activity), which are constitutively active in DMD muscles	Reduction of necrosis and fibrotic and adipose tissue deposition	No restoration of dystrophin expression	Phase III ongoing	[54, 55, 73]
Idebenone (Catena/Raxone) *Santhera* *Pharmaceuticals*	Chemical short‐chain benzoquinone; potent antioxidant and lipid peroxidation inhibitor at mitochondrial level	Expected cardioprotection and improvement of muscle performance and respiratory functions	No restoration of dystrophin expression	Phase III ongoing	[59, 60, 61]
Tadalafil and Sildenafil *Eli Lilly and Company*	PDE5 inhibitor induces vasodilatation through cGMP signalling activation	Expected improvement of muscle blood flow during physical exercise	No restoration of dystrophin expression Little evidence of benefits	Phase III completed	[56, 57, 76]
Vamorolone (VBP15) *ReveraGen BioPharma*	Glucocorticoid‐like oral drug with anti‐inflammatory and membrane‐stabilizing properties	Reduction of muscle inflammation No glucocorticoid‐associated side effects	No restoration of dystrophin expression	Phase II ongoing	[72]

cGMP, cyclic guanosine monophosphate; HDAC, histone deacetylase; nmDMD, nonsense mutation Duchenne muscular dystrophy; PDE5, phosphodiesterase‐5.

4

More recently, researchers are trying to useCRISPR/Cas9 genome editing system to remove mutated exons from the *DMD* gene.^85^, ^86^ This method uses small guide RNAs coupled with target‐specific double‐strand DNA endonuclease making possible targeted gene disruption, replacement or modification.^87^ The CRISPR/Cas9 approach has shown ability to rescue dystrophin expression in DMD patient‐derived iPSC in vitro, and in muscles of experimental models of DMD in vivo.^88^ Similar to AON, application of CRISPR/Cas9 method to DMD patients requires a personalized setting depending on the specific mutation. Other limitations for the use of CRISPR/Cas9 as a DMD treatment are represented by possible off‐targeting and activation of the host immune response.^89^

Such an extremely varying scenario in the therapeutic approaches to DMD is the result of the difficulty and, at the same time, the intense effort to find a cure for this pathology. Thus, the current gold standard therapy for DMD patients remains the use of anti‐inflammatory steroids (eg, Prednisone and Deflazacort), which improve the quality of life reducing loss of muscle strength and functionality and loss of ambulation, and delaying respiratory failure.^45^, ^90^ However, corticosteroids have shown limited activity and cause several adverse effects, including gain of weight, reduction of bone mineral density, cushingoid appearance, behavioural changes, adrenal suppression, susceptibility to infection, hypertension and metabolic disorders,^91^, ^92^ so that alternative anti‐inflammatory compounds such as the NF‐κB inhibitor, VBP15, are also under investigation^93^ (Table 3).

## 5 DO PORCINE SERTOLI CELLS REPRESENT AN OPPORTUNITY FOR DUCHENNE MUSCULAR DYSTROPHY?

**Figure 1:**
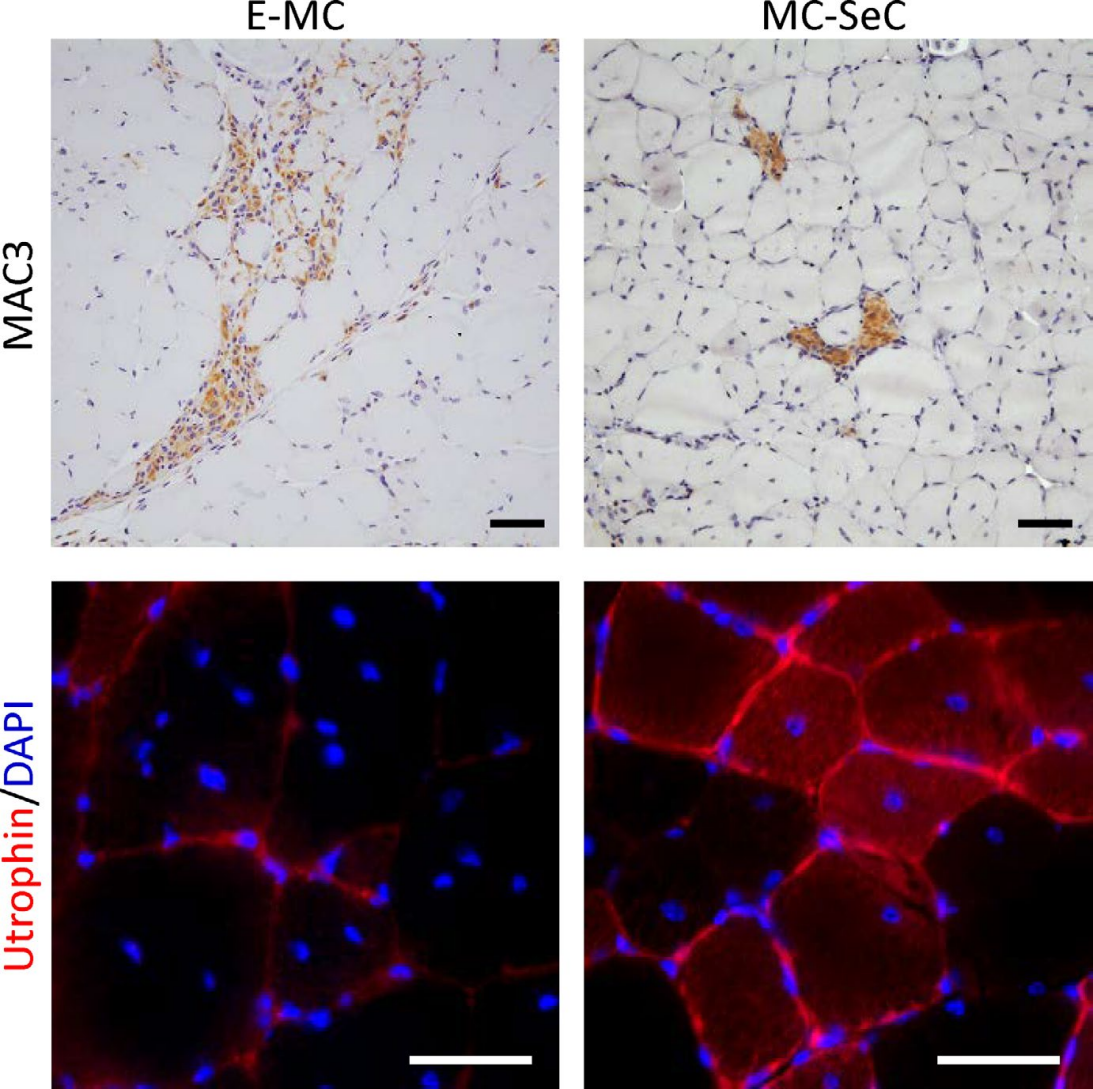
Intraperitoneal injection with microencapsulated Sertoli cells (MC‐SeC) in dystrophic mice results in reduced inflammation and re‐expression of utrophin in muscles. *Tibialis anterior* muscles of acute phase (4‐wk‐old) *mdx* mice analysed for the presence of the activated macrophage marker, MAC3, by immunohistochemistry (anti‐MAC3 antibody, clone M3/84; BD Biosciences) (upper panel) and the expression of utrophin by immunofluorescence (anti‐utrophin antibody, clone 8A4; Santa Cruz Biotechnology) (lower panel) 3 wk after ip injection with MC‐SeC or the same amounts of empty microcapsules (E‐MC). Note the significant reduction of inflammatory infiltrate (ie, MAC3‐positive areas) and the positivity for utrophin at the sarcolemma in muscles of MC‐SeC–treated mice. Original magnification, 20× (upper images) and 40× (lower images)

Having the peculiar secretory properties of SeC in mind, we treated acute and chronic dystrophic, *mdx* mice with a single ip injection of porcine MC‐SeC (equivalent amount, 1.0 × 10^6^ SeC/gram of body weight) in the absence of any pharmacologic immunosuppression, and found a rapid amelioration of muscle morphology and functionality.^94^, ^95^ After 3 weeks from injection, muscles of treated mice showed ~80% reduction of the inflammatory infiltrate (as assed by evaluation of activated macrophages marker, MAC3; Figure 1), ~60%‐70% reduction of fibrous tissue deposition, and over 90% reduction of necrotic myofibres in *Tibialis anterior* muscle. At the same time, MC‐SeC–treated *mdx* mice showed increased muscle performance and resistance to exercise‐induced muscle damage. Indeed, treated mice ran similar distances in a similar time to untreated WT mice, recovering ~80% of dystrophic‐dependent deficit, and showed a ~70% reduction of damaged (Evans blue dye–positive) myofibres after treadmill exercise tests. The anti‐inflammatory effect of MC‐SeC was observed already 1 week after injection, when ~70% reduction of macrophages infiltrating muscle tissue could been observed. At the same time, these macrophages showed a tissue‐repairing (M2) phenotype, as suggested by their reduced expression of inflammatory cytokines (ie, IL‐6, IL‐12 and IFN‐γ) and up‐regulation of the anti‐inflammatory IL‐10 and the M2 markers, arginase 1, CD163 and CD206.^94^ Interestingly, a single ip injection of MC‐SeC conferred benefits (ie, significantly reduced necrosis, inflammatory infiltrate, and fibrotic and adipose tissues deposition) in long‐term (5 months) analysis to the diaphragm, the muscle that accumulates damage over time in the *mdx* animal model. No signs of immune response against injected MC‐SeC could be detected at this time.^94^

Another important aspect of SeC treatment is that SeC release heregulin β1, which is a major inducer of the expression of utrophin in muscle cells.^96^ Heregulin β1 acts by binding to erbB/HER receptor, resulting in intracellular ERK activation and subsequent binding of the *ets*‐related GABPα/β transcription factor complex to the utrophin‐A promoter.^97^ Three weeks after ip injection of MC‐SeC, a ~2.8‐fold increase in utrophin expression was observed in muscles of *mdx* mice,^94^ which is similar to that reported after 3 months of repeated ip injections each other day of the active domain of heregulin β1 (Aa 176‐246) in the same experimental model.^98^ In both cases, utrophin was found localized at the sarcolemma, a condition necessary for the protein to mimic the role of dystrophin (Figure 1).

Microcapsules containing SeC–based protocol resulted efficacious also in pre‐symptomatic (2‐week‐old) and in chronic (12‐month‐old) *mdx* mice.^95^ In the diaphragms of these latter mice, a significant reduction of adipose and fibrous tissue deposition (~43% and ~58% reduction, respectively), macrophage infiltrate (~70% reduction of MAC3‐positive areas) and damaged myofibres (more than 80% reduction of EBD‐positive myofibres) were observed 3 weeks after ip injection of MC‐SeC.^95^

The above reported results about the use of MC‐SeC have opened an additional avenue in the scenario of DMD treatment. Intraperitoneally injected MC‐SeC act as a micro‐biofactory that from the peritoneal cavity of dystrophic animals release factors into the bloodstream thus being able to reach every muscle where they exert a double effect: (a) an anti‐inflammatory effect (as expected) due to immunomodulatory factors; and (b) the induction of utrophin expression due to the SeC‐secreted heregulin β1.^94^ These two effects are independent from each other but cooperate to ameliorate muscle morphology activating a positive loop that leads to reduction of necrosis, fibrosis and adipose tissue deposition, finally culminating in rescue of muscle architecture and performance. Indeed, the use of an anti‐heregulin β1 antibody nullified the SeC‐dependent induction of utrophin in *mdx* muscles. However, anti‐heregulin β1 antibody had no significant effects on the reduction of the inflammatory infiltrate, suggesting that this latter effect is under the control of anti‐inflammatory factors. On the converse, only a partial loss of anti‐necrotic effects could be observed on myofibres of *mdx* mice treated with MC‐SeC in the presence of anti‐heregulin β1 antibody, due to a balance between increased damage extent of (dystrophin‐negative/utrophin‐negative) myofibres and the effects of anti‐inflammatory factors.^94^

Several considerations give particular relevance to the porcine MC‐SeC–based therapeutic approach (Table 4). On the side of the biomaterial used, (a) highly biocompatible, clinical grade alginate (endotoxin content less than 0.5 EU/mL, as required for human transplants) was used for the production of microcapsules^15^, ^16^, ^17^, ^18^, ^19^, ^20^, ^21^, ^22^, ^94^, ^95^, ^101^, ^102^, ^103^, ^105^; (b) alginate‐based microcapsules have shown long‐term survival and activity of entrapped cells^22^, ^94^, ^99^, ^100^ with porcine IGF‐1 being detected in the serum of mice treated with porcine‐derived SeC up to 1 year after injection^22^; and (c) alginate‐based microcapsules containing human pancreatic islets have been employed in a phase I clinical trial in which they were transplanted ip in non‐immunosuppressed type 1 diabetic patients with no undesired effects reported.^101^, ^102^, ^103^ On the side of SeC, (a) SeC were purified from testis of SPF (specific pathogen free) piglets, that is, animals suitable for engraftment in humans; 104 (b) MC‐SeC were injected ip in spontaneous type 2 diabetes non‐human primates (rhesus macaques) resulting in reduction of plasma glucose and B lymphocytes, and absence of adverse effects^105^; (c) neonatal porcine SeC were inserted together with pancreatic islets subcutaneously in a porous chamber in the abdominal wall of young diabetic patients, in the absence of immunosuppressive treatment, and half patients significantly diminished their insulin doses with no complications reported in a 7‐year follow‐up.^106^, ^107^

**Table 4:** Advantages and disadvantages of the MC‐SeC approach to DMD

Approach	Advantages	Disadvantages	References
Intraperitoneal injection of MC‐SeC	Independent from DMD gene mutation	Need for xenogeneic source of SeC	94, 95
	All muscles interested thanks to the systemic release of SeC‐derived factors	Caution for PERV presence in pig‐derived SeC, especially in immunosuppressed patients	[108, 109, 110, 111, 112, 113]
	Combinatorial approach (ie, anti‐inflammatory effect, induction of utrophin expression and release of trophic factors)	‐	94, 95
	No need for immunosuppression	‐	15, 18, 19, 20, 21, 22, 94, 95, 106
	Single ip injection not requiring incision of the abdominal wall	‐	15, 18, 19, 20, 21, 22, 94, 95, 106
	No undesired effects reported in several pre‐clinical settings (including non‐human primates)	‐	[15, 18, 19, 20, 21, 22, 94, 95, 102]
	SeC (non‐encapsulated) already used in clinical trials; no undesired effects reported	‐	[106, 107]
	Alginate‐based microcapsules (containing cells other than SeC) already used in clinical trials; no undesired effects reported		[99, 100, 101]

MC‐SeC, microencapsulated Sertoli cells; PERVs, porcine endogenous retroviruses; SeC, Sertoli cells.

The use of pig cells, tissues and organs meets the general need to satisfy the increasing request for transplantation by humans who do not find sufficient availability among members of their species. Pigs represent suitable animals for human xenotransplantation because they share a similar organ physiology and size, for the relatively low costs of breeding and for the possibility to be genetically modified. One concern in xenotransplantation using pigs as a donor species is represented by porcine endogenous retroviruses (PERVs). This is because PERVs are present in almost all strains of pigs and cannot be removed even if pigs are being raised in sterile conditions. PERVs are inactive and harmless in pigs; however, transplantation into humans could activate the viruses and lead to human diseases with the risk of spreading to the entire community.^108^ However, although PERV can infect human cells in vitro, (a) transmission of PERV was not observed in animals (including non‐human primates) inoculated with PERV preparations or in pre‐clinical xenotransplantations (reviewed in 109); (b) patients xenotransplanted with porcine islets and SeC showed no PERV infection in their white blood cell DNA in long‐term clinical follow‐up^110^; and (c) studies of around 200 people worldwide who had been transplanted with pig tissue or had their blood pass through pig cells have shown no evidence of infection of porcine origin, and neither antibodies against PERV nor provirus integration in patients’ blood cells was observed.^111^ The reason why PERVs are not transmitted is that they probably are not released from the transplants or they are neutralized by the host cellular defence and immune system.^109^

However, porcine pancreatic islets were demonstrated to produce PERV and infect NOD/SCID (non‐obese diabetic, severe combined immunodeficiency) mice after transplantation, suggesting that PERV infection is a risk to take into account when pig xenotransplantation involves immunocompromised subjects.^112^ In this regard, it is noteworthy that PERV‐inactivated pigs have been recently generated *via* somatic cell nuclear transfer using a cell line in which PERVs were inactivated by CRISPR/Cas9 technology.^113^

Among the factors secreted by SeC are mitogenic factors that could potentially sustain cell growth and induce the formation of tumour masses in the recipient. However, this event has not been reported in any study involving the use of SeC, including the above‐mentioned clinical study.^110^ Probably, this is because SeC secrete a cocktail of molecules whose global effect results from the combination of all factors rather than being the sum of each single factor activity.

Another issue about the use of SeC is related to their immunosuppressive effect, representing a potential problem in case of lifelong clinical applications. However, results from several pre‐clinical and clinical studies suggest that SeC have an immunomodulatory rather than immunosuppressive effect.^2^, ^5^ Interestingly, SeC responded to viruses and bacteria eliciting an inflammatory response *via* the recruitment of Toll‐like receptors expressed on SeC surface and subsequent release of proinflammatory cytokines and chemokines.^114^

Although data obtained in macaques 105 and data from the experimentation in^106^ support the safety of the use of SeC in humans, absence of SeC‐induced tumour formation and the immunomodulatory vs immunosuppressive role of SeC should be addressed definitively in animal models.

## 6 CONCLUSIONS

Duchenne muscular dystrophy is a lethal muscular dystrophy affecting 1 in 3600‐5000 male live births worldwide.^115^ The progressive muscle degeneration subsequent to lack of dystrophin creates a condition of chronic inflammation that culminates in the progressive substitution of myofibres with fibrous and adipose tissues, impaired locomotion and premature death. Several intrinsic properties of the DMD pathology have nullified the huge effort to find a cure so far. In addition, many suggested therapeutic treatments have immunosuppression as a necessary co‐treatment, which means adding problem to problem, especially in a lifelong perspective. Therefore, investigation is still particularly active on DMD, and combinatorial therapeutic approaches might be envisaged.

**Figure 2:**
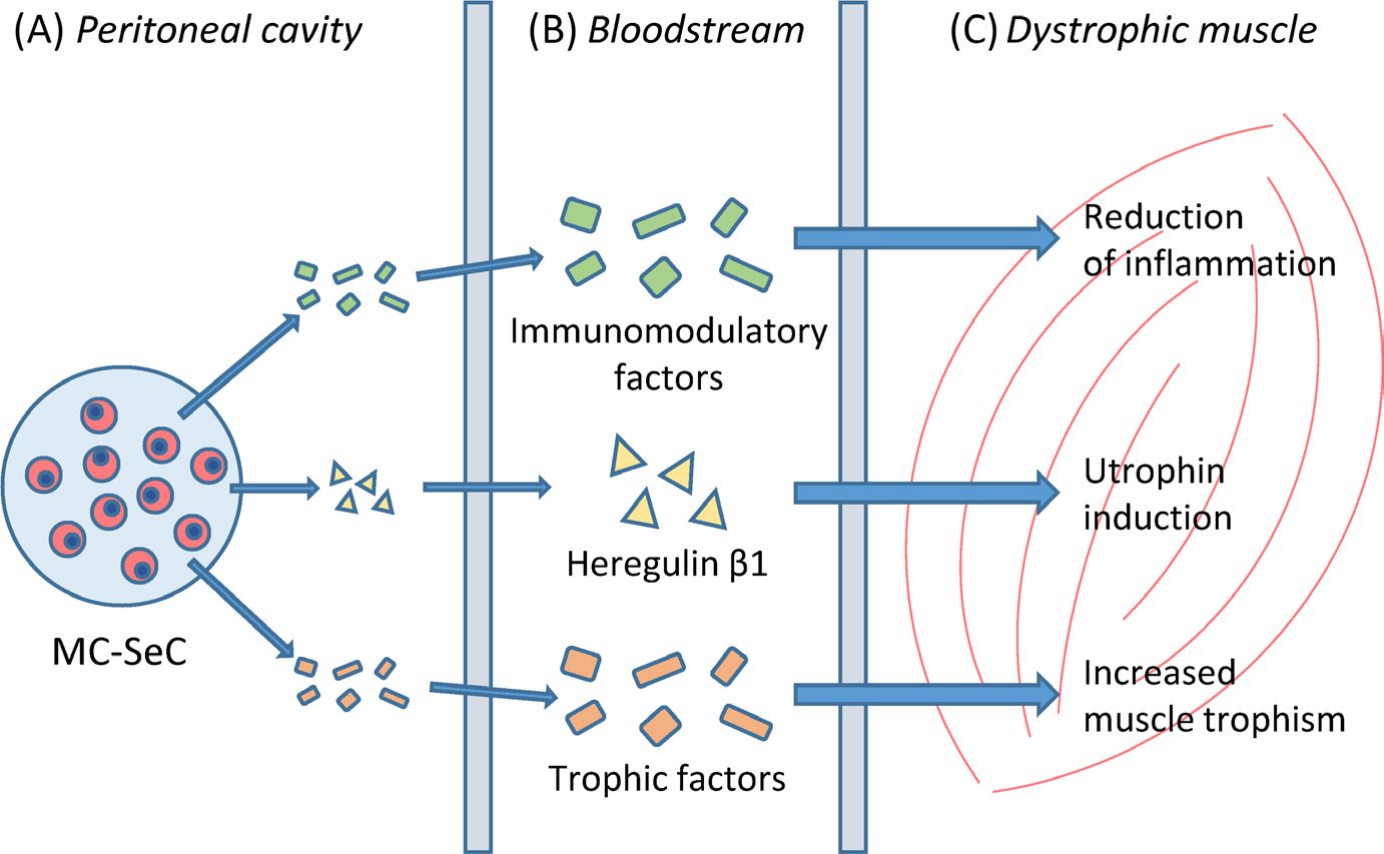
Schematics of the microencapsulated Sertoli cells (MC‐SeC) therapeutic approach. Once injected into the peritoneal cavity of dystrophic mice, MC‐SeC release a cocktail of factors including immunomodulatory factors, heregulin β1 and trophic factors (A). From the peritoneum, SeC‐released factors enter the systemic circulation (B) through which they can reach all skeletal muscle compartments (including cardiac muscle). At muscle tissue level, SeC‐released factors reduce the inflammatory response, induce utrophin expression and favour muscle trophism (C), thus recovering muscle morphology and functionality. It is noteworthy that thanks to the immunomodulatory properties of SeC, the procedure does not require pharmacological immunosuppression

Intraperitoneal injection of MC‐SeC translates into the release into the bloodstream of a cocktail of factors of which at least two components (ie, anti‐inflammatory factors and heregulin β1) are independently active on dystrophic muscles,^94^ representing in this sense a combinatorial approach per se (Figure 2). Interestingly, both mechanisms involved in MC‐SeC treatment are known to be very efficacious in DMD patients: the extinction of the inflammatory response and the induction of expression of the dystrophin paralogue, utrophin, at the sarcolemma. Based on the biology of SeC, it cannot be excluded that other factors secreted by this cell type (eg, IGF‐1) may additionally concur to the amelioration of dystrophic muscle morphology (Figure 2), which deserves further investigation.

Encapsulation represents an additional point of force of the proposed approach as encapsulation encloses the cells in a defined space, avoiding cell migration throughout the host body, and potentially allowing the recovery of injected cells if the injection is performed in a confined region of the body, such as the peritoneal cavity.

It is noteworthy that treatment with MC‐SeC has the advantage to be a universal approach to treat DMD since it is potentially applicable to the entire cohort of DMD patients regardless of the kind of mutation. Moreover, it is potentially applicable in general to myopathies characterized by chronic inflammation or immune dysregulation, such as autoimmune myositis.

Elucidation of several aspects in the near future, including the biological status of SeC inside the microcapsules over time, the dose‐response of MC‐SeC, potential direct effects of SeC on muscle precursor cells, the analysis of the mechanism(s) underpinning the anti‐inflammatory effect of SeC, and the demonstration of the safety of MC‐SeC in long‐term treatments will accelerate the application of this novel therapeutic approach to DMD patients.

## CONFLICT OF INTEREST

The authors declare no conflict of interest. The founding sponsors had no role in the design of the study; in the collection, analyses or interpretation of data; in the writing of the manuscript; and in the decision to publish the results.
